# Prevalence and genotype distribution of HPV combined with cervical pathological results in women from Sichuan, China: A cross‐sectional study based on post‐vaccination period 2019 to 2023

**DOI:** 10.1002/cam4.70148

**Published:** 2024-08-27

**Authors:** Bangzhu Mo, Yuanxin Ye, Maowen Yu, Xianli Tong, Hongmei Cao, Chunmei Du, Jiangrong Luo, Chunbao Xie

**Affiliations:** ^1^ Department of Laboratory Medicine Jintang First People's Hospital, Sichuan University, West China Hospital, Jintang Hospital Chengdu Sichuan China; ^2^ Department of Laboratory Medicine West China Hospital Chengdu Sichuan China; ^3^ Department of Obstetrics and Gynecology Jintang First People's Hospital, Sichuan University, West China Hospital, Jintang Hospital Chengdu Sichuan China; ^4^ Department of Pathology Jintang First People's Hospital, Sichuan University, West China Hospital, Jintang Hospital Chengdu Sichuan China; ^5^ Department of Anesthesiology Sichuan Provincial People's Hospital, University of Electronic Science and Technology of China Chengdu Sichuan China; ^6^ Department of Laboratory Medicine and Sichuan Provincial Key Laboratory for Human Disease Gene Study Sichuan Provincial People's Hospital, University of Electronic Science and Technology of China Chengdu Sichuan China

**Keywords:** age‐specific, cervical cancer, cytological abnormal, genotype, HPV, vaccination

## Abstract

**Background:**

Human papillomavirus (HPV) screening and vaccination exert efficacy in controlling the progression of cervical cancer. Thus, examinations into HPV prevalence, age‐stratified specificity, genotype distribution, and their correlation with pathological outcomes can furnish robust evidence for customizing high‐quality population screening and management.

**Methods:**

A cohort of 17,923 women attending clinics in the Jintang area, Sichuan, from January 2019 through August 2023 were enrolled in the study. Genotyping of HPV was conducted using real‐time polymerase chain reaction (RT‐PCR). The epidemiology and the relationship between HPV infection and histologic/cytologic abnormalities were subjected to analysis.

**Results:**

HPV infection was identified in 4387 women. The outpatient group exhibited a significantly higher HPV infection rate compared to the healthy examination group (26.5% vs. 17.5%, *p* < 0.05). The distribution of infection rates across different age groups exhibited a U‐shaped pattern, with the highest infection rate in the group ≤20 years of age, succeeded by those >60 years of age. The 31–40 age group demonstrated the lowest prevalence of infection, but upon infection, its prevalence of the precancerous lesion CIN2‐3 reached a maximum of 29.0%, constituting a novel finding. The most prevalent genotype was HPV52, followed by HPV16, 58, 53, 68, and 18. In the cytologic and histologic abnormalities group, the most common types were HPV52, 16, and 58. HPV16 predominantly appeared in high‐grade intraepithelial neoplasia and carcinoma in situ, constituting over 60% of cases. While HPV type 52 was not individually detected in cervical cancer cases. And some other non‐vaccine‐covered HPV subtypes also showed high prevalence in Sichuan. The single infection rates of NH9‐HPV (high‐risk HPV subtypes covered by the non‐nine‐valent vaccine) in CIN2‐3 and cervical cancer patients were 6.5% and 2.6%, respectively. Among them, HPV51, HPV53, HPV59, and HPV35 exhibited a significant preponderance, which even higher than HPV45 and HPV31 covered by the nine‐valent vaccine types. And in NL9‐HPV (low‐risk HPV subtypes covered by the non‐nine‐valent vaccine), HPV42 accounted for the highest percentage in CIN2‐3. A similar decreasing trend was observed in annual infection rates in the healthy examination population and in the 31–40 and 51–60 age groups, while the ≤20 age group showed an increase. Regarding type‐specificity, HPV16 and HPV58 exhibited the most rapid declines.

**Conclusion:**

This study furnishes the latest insights into the characteristics of HPV infection rate, age distribution, and genotype prevalence in Sichuan.

## 1 INTRODUCTION

Cervical cancer ranks as the fourth most prevalent cancer among females globally,^1^ posing a significant threat to women's health. The incidence of cervical cancer has experienced a noteworthy surge in the past. The absolute number of global cervical cancer cases estimated by GLOBOCAN has increased from 529,000 cases in 2008 to 570,000 cases in 2018.^2^ In China, the cervical cancer cases have increased by 3.8% and deaths by 4.4% over the past few years, and the cancer burden is anticipated to persist in its upward trajectory over the coming decade.^3^ According to the Global Cancer Statistics of 2020,^4^ there are approximately 604,200 new cases and 310,000 deaths worldwide, with nearly 90% of fatalities occurring in developing nations.^5^ As one such developing nation, China is projected to witness 111,820 new cases of cervical cancer and 61,579 deaths in 2022.^6^ Consequently, the prevention of cervical cancer and its precursors and the reduction of mortality in cervical cancer patients stand as pressing concerns.

The persistent high‐risk human papillomavirus (HPV) infection is fundamental to the development of precancerous lesions and cervical cancer.^7^ In accordance with the International Agency for Research on Cancer (IARC) classification, twelve HPV types (HPV16, HPV18, HPV31, HPV33, HPV35, HPV39, HPV45, HPV51, HPV52, HPV56, HPV58, and HPV59) are designated as Group 1 human carcinogens (Group 1A), with HPV68 classified as a probable carcinogen in Group 2A, and HPV26, HPV53, HPV66, HPV67, HPV70, HPV73, and HPV82 as probable carcinogens in Group 2B.^8^ Studies have elucidated that 98.7% of cervical cancers can be attributed to one of the 13 most common HPV types mentioned above (Groups 1A and 2A) and seven other HPV types (Group 2B).^9^ Furthermore, HPV is implicated in various premalignant and malignant lesions, encompassing penile, vaginal, anal, vulvar, oropharyngeal, and pulmonary cancers, as well as condyloma acuminata.^10^, ^11^

Cervical cancer presently stands as the sole tumor with a well‐defined etiology that can be prevented through screening and vaccination.^12^, ^13^ In 2023, China released the Action Plan for Accelerated Elimination of Cervical Cancer (2023–2030),^14^ which proposes an expected that screening 70% of women by the age between 35 and 65 using a high‐performance test and treating at least 90% of identified precancerous lesions and invasive cancers by 2030. It also recommends that the starting age for screening in average‐risk women be 25–30 years old. However, at present, the practical problems of low coverage of cervical cancer screening, lack of advanced technology for large‐scale screening, limited community service capacity, and poor accessibility of HPV screening are still exist in China. Increasing the rate of HPV prophylactic vaccination of girls aged 9–14 years is also a key focus of the action plan. Meanwhile, due to public issues such as the lack of implementation of the national immunization program, insufficient supply of HPV vaccine, and concerns about the effectiveness and safety of the vaccine, the vaccination rate is only 3% for the women in China, and 1.9% for the girls aged 9–14 years according to the survey data from 2021.^15^, ^16^ China still far away from the WHO's goal of “90‐70‐90” (90% of girls fully vaccinated against HPV by age 15, 70% of women receiving high‐performance testing by age 35, and 90% of women receiving treatment by age 45).^17^

Recent clinical data underscore the significance of parameters such as the prevalence of HPV infection, genotype distribution, and their correlation with histologic/cytologic triage results in assessing the risk of cervical cancer in women.^12^, ^18^ These parameters markedly enhance the promptness and efficacy of local clinical diagnoses of cervical cancer. The manner of progression to cervical cancer differs depending on the type of HR‐HPV: HPV16 is characterized by gradual carcinogenesis, HPV18 is difficult to detect in precancerous lesions, and HPV52, 58 tends to stay in cervical intraepithelial neoplasia (CIN) status.^19^ Since 2018, three vaccines had been available in the Chinese domestic market: 2vHPV, 4vHPV and 9vHPV. Vaccination has induced alterations in the distribution of certain HPV genotypes.^20^ The prevalence of HPV types covered by the vaccine may be relatively reduced, whereas the infection rates of other non‐vaccine‐covered oncogenic genotypes may be relatively increased.^21^, ^22^ It suggests that it is necessary to pay close attention to some non‐target genotypes of HPV vaccines along with the promotion of existing prophylactic HPV vaccination.^22^, ^23^ Nevertheless, the prevalence and effect of HPV subtypes not covered by the nine‐valent vaccine in patients with cervical intraepithelial neoplasia and cervical cancer are rarely studied, as well as the trends in HPV genotype changes in the post‐vaccine era. Concurrently, regional and temporal differences in HPV infection prevalence exist due to variations in population demographics, geographical location, socioeconomic status, age, ethnicity, and lifestyle.^24^, ^25^, ^26^ Therefore, there is a necessity for site‐specific epidemiologic studies on HPV within specific populations and its relationship with clinical characteristics.

In this study, it conducts a comprehensive examination of data related to genotypes (including HPV subtypes covered by the nine‐valent vaccine and non‐nine‐valent vaccine), 5‐year prevalence trends, age stratification, and pathological findings of HPV infection in women in Sichuan, Western China, spanning from 2019 to 2023. The objective is to elucidate the burden of HPV among women with different cervical pathological states in the region, thereby furnishing a scientific foundation for optimizing cervical cancer screening and vaccination strategies in the post‐vaccine era.

## 2 MATERIALS AND METHODS

### 2.1 Study subjects

The study encompassed outpatient, inpatient, and physical examination women who underwent routine HPV screening from January 2019 to August 2023 at the Jintang First People's Hospital, Sichuan Province, China. The outpatients visited the hospital for various reasons, including: pelvic inflammatory disease, vaginitis, cervicitis, genital warts, and cervical intraepithelial neoplasia. All participants underwent HPV genotype testing. Exclusion criteria comprised total hysterectomy or cervical resection, systemic infection or autoimmune disease, and other cancers. Ethical approval for the study was obtained from the Ethics Committee of the Jintang First People's Hospital (approval number: 20231017036) and informed consent is waived.

### 2.2 Cervical specimen collection

Cervical samples were acquired in this study using a specialized cervical exfoliative cell collector. HPV detection was performed using a cytobrush, and the brush's head was placed in a small bottle containing preserving fluid. The specimens were promptly stored at 4°C and subsequently sent to our clinical laboratory within 24 hours for HPV analysis.

### 2.3 HPV DNA detection and typing methods

Multiplex fluorescent PCR kits (including extraction and amplification) (Chaozhou Kaipu Biochemistry Limited Corporation, China) was utilized in this study following the manufacturer's protocol. Genotyping tests could identify 17 high‐risk HPV (HR‐HPV) types: HPV16, 18, 31, 33, 35, 39, 45, 51, 52, 53, 56, 58, 59, 66, 68, 73, and 82, and 6 low‐risk HPV (LR‐HPV) types: HPV6, 11, 42, 43, 44, and 81. Intracellular control DNA ensured sample quality throughout the experiment, and external negative and positive controls were employed for quality control, ensuring that the results adhered to laboratory standards.

### 2.4 Thin‐layer liquid‐based cytology test

Cervical sections were prepared using the Auto‐CytoThin II cytology system. Following the Bethesda system, specimens were categorized as negative for intraepithelial lesion or malignancy (NILM), atypical squamous cells of undetermined significance (ASCUS), atypical gland cell (AGC), low‐grade squamous intraepithelial lesion (LSIL), and high‐grade squamous intraepithelial lesion (HSIL).

### 2.5 Colposcopy and histologic diagnosis

Colposcopy was performed on individuals with abnormal cytologic results, and cervical tissue biopsy was conducted on HPV‐positive patients with clearly visible cervical abnormalities. Disease classification was determined based on the highest level of cervical histopathologic findings, evaluated by two experienced pathologists. Study results were categorized as No lesions, cervical intraepithelial neoplasia grade 1 (CIN1), CIN2, CIN3, cervical cancer (CA).

### 2.6 Statistical analysis

All statistical analyses were conducted using SPSS 26.0 (Chicago, USA) statistical software. HPV prevalence and age‐stratified prevalence rates, along with their exact binomial 95% confidence intervals (CIs), were calculated. Pearson's χ^2^ test was employed to assess the significance of differences between designated groups. Infection rate comparisons between age subgroups utilized the Bonferroni multiple comparison correction method to adjust for Type I error in multiple comparisons,^27^, ^28^ and *p* < 0.05 is the displayed value corrected by SPSS. All analyses were two‐sided, and *p* < 0.05 was interpreted as significant.

## 3 RESULTS

### 3.1 Overall prevalence of HPV infection

A total of 17,923 females were included in this study, with a mean age of 39.40 ± 11.63 (range 13–86). Among them, 4387 tested positive for HPV, resulting in an overall infection rate of 24.5%. The overall infection rates exhibited statistically significant differences across the years 2019–2023 (χ^2^ = 30.3, *p* < 0.001). Notably, there was a significant decrease in infection rates observed in 2023 compared to data from prior to 2021. Concurrently, a downward trend in overall HPV infection rates transpired over the 5‐year period (see Table 1).

**TABLE 1:** Overall prevalence of human papillomavirus (HPV) infection and the prevalence between outpatient and physical examination groups.

Year	Outpatient HPV+	Healthy examination HPV+	Total HPV+	*p*‐Value
	*N*, % (95% CI)	*N*, % (95% CI)	*N*, % (95% CI)	
2019	2679,26.8 (25.2–28.6)	337,19.0 (14.9–23.6)	3019,26.0 (24.4–27.6)	0.002
2020	2324,27.3 (25.5–29.1)	432,22.0 (18.2–26.2)	2759,26.5 (24.9–28.2)	0.022
2021	4345,26.6 (25.3–27.9)	870,18.9 (16.3–21.6)	5220,25.3 (24.2–26.5)	<0.001
2022	2856,25.3 (23.7–26.9)	970,16.4 (14.1–18.9)	3844,23.0 (21.6–24.3)	<0.001
2023	1673,26.4 (24.3–28.5)	1382,15.8 (13.9–17.8)	3081,21.6 (20.2–23.1)	<0.001
Total	13,877,26.5 (25.7–27.2)	3991,17.5 (16.4–18.8)	17,923,24.5 (23.8–25.1)^a^, ^b^, ^c^	<0.001

^a^ The proportion of total infection in 2023 was significantly different from that in 2019 (χ^2^ = 16.2, *p* < 0.001).

^b^ The proportion of total infection in 2023 was significantly different from that in 2020 (χ^2^ = 19.0, *p* < 0.001).

^c^ The proportion of total infection in 2023 was significantly different from that in 2021 (χ^2^ = 14.6, *p* < 0.001).

### 3.2 Prevalence of HPV infection in outpatient and physical examination groups

Excluding the smaller cohort of hospitalized cases (*n* = 55), the participant population was stratified into an outpatient group and a physical examination group, comprising 13,877 and 3991 cases, respectively. The mean age of outpatient was 38.32 ± 11.77. And the physical examination groups 42.95 ± 10.13. There was no significant difference between the two groups (*p* > 0.05). The prevalence of HPV infection in the outpatient group was notably higher than that in the physical examination group (26.5% vs. 17.5%, *p* < 0.001). The prevalence of HPV infection displayed a significant difference (*p* < 0.05) between both outpatient and physical examination groups from 2019 to 2023 (Table 1). The infection rate in the physical examination population demonstrated a declining trend year by year, while the infection rate in the outpatient population decreased during 2020–2022 and exhibited an upward trend in 2023.

### 3.3 Prevalence of HPV genotypes

**FIGURE 1:**
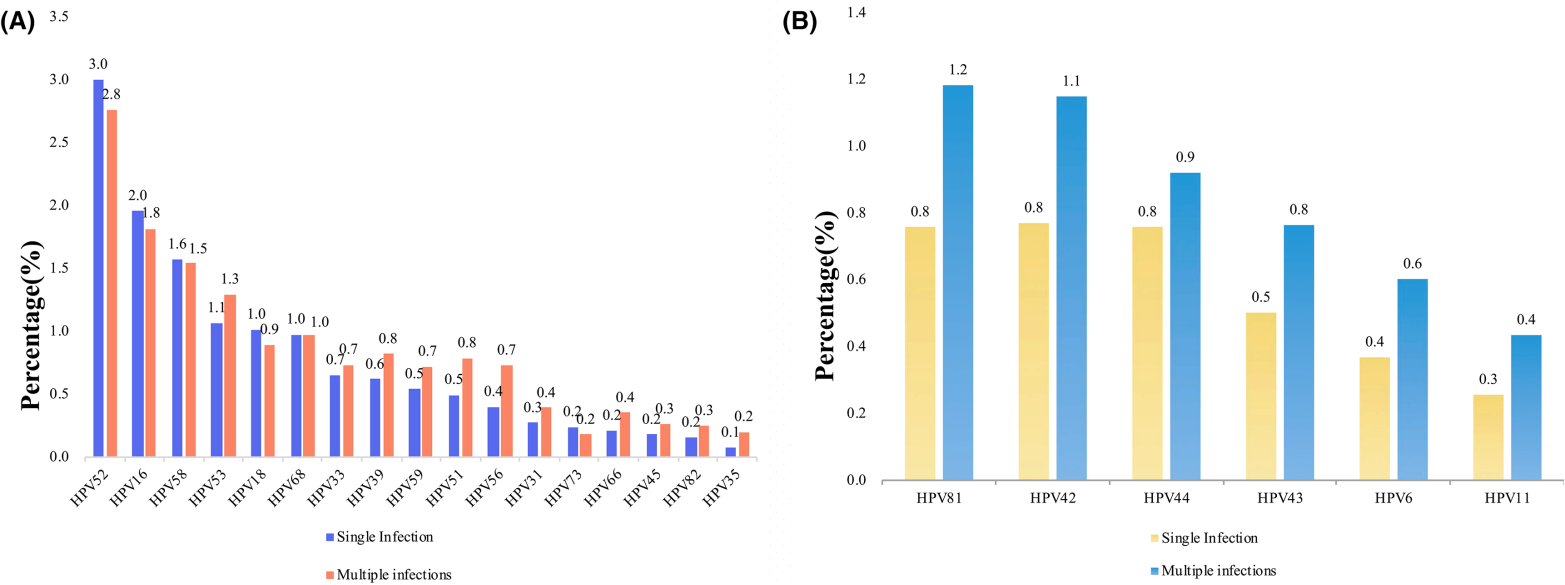
The prevalence of different HPV genotypes. (A) The prevalence of HR‐HPV genotypes; (B) The prevalence of LR‐HPV genotypes. HPV, human papillomavirus.

The findings indicate that the six predominant high‐risk (HR‐HPV) genotypes, ranked in descending order, were HPV52, HPV16, HPV58, HPV53, HPV68, and HPV18, with infection rates of 5.8%, 3.8%, 3.1%, 2.4%, 2.0%, and 1.9%, respectively (refer to Figure 1A). Conversely, the six prevalent low‐risk (LR‐HPV) genotypes were HPV81, HPV42, HPV44, HPV43, HPV6, and HPV11, exhibiting prevalence rates of 1.9%, 1.9%, 1.7%, 1.3%, 1.0%, and 0.7%, respectively (Figure 1B). And in the context of HR‐HPV, HPV52, HPV16, HPV58, HPV18, and HPV73 primarily presented as mono‐infections, while HPV53, HPV33, HPV39, HPV59, HPV51, HPV56, HPV31, HPV66, HPV45, HPV82, and HPV35 predominantly occurred as multiple infections (see Figure 1A). Among the LR‐HPV types, HPV81, HPV42, HPV44, HPV43, HPV6, and HPV11 predominantly exhibited multiple infections. Notably, the prevalence of HPV81, HPV42, HPV44, and HPV43 was higher than HPV6 and HPV11, both in terms of single and multiple infections (see Figure 1B).

**FIGURE 2:**
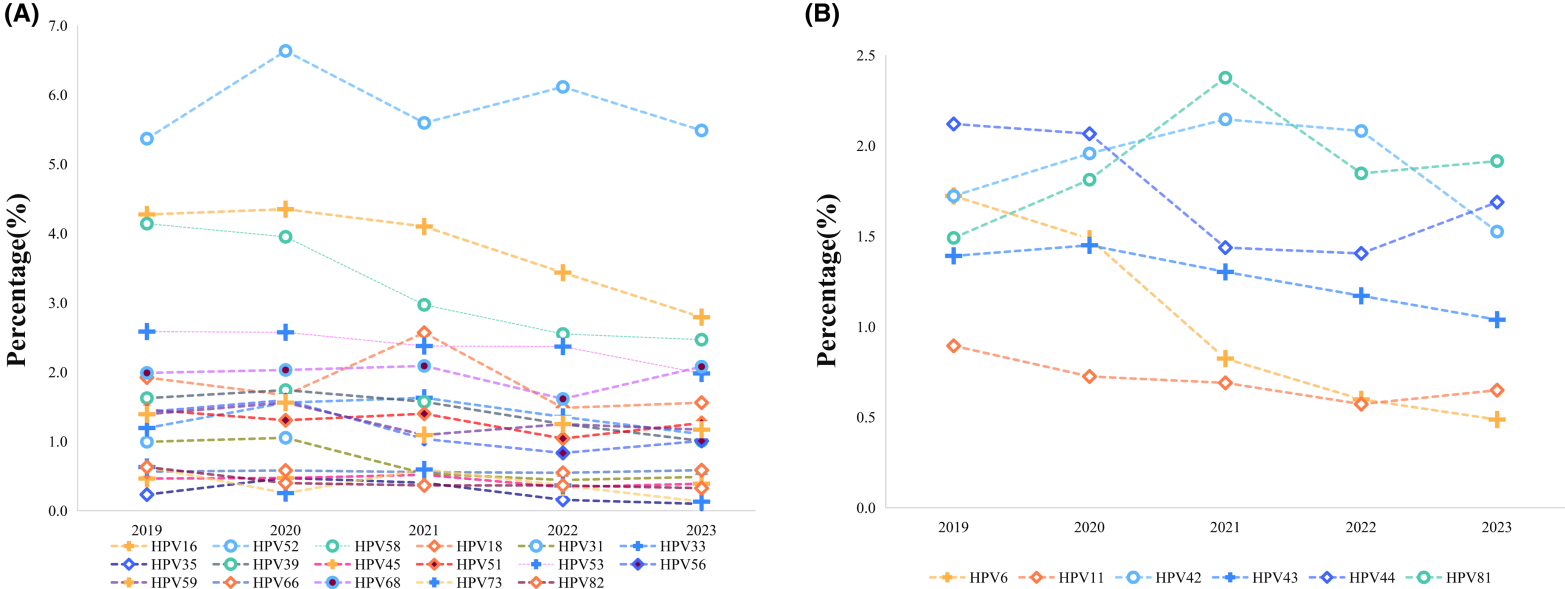
The trends of prevalence of different HPV genotypes from 2019 to 2023. (A) The proportion of HR‐HPV genotypes; (B) The proportion of LR‐HPV genotypes. HPV, human papillomavirus.

As demonstrated in Figure 2A, the HR‐HPV genotypes, including HPV16, HPV31, HPV35, HPV39, HPV56, HPV58, and HPV73, exhibited a diminishing trend in infection rates over the five‐year period. Notably, HPV58 demonstrated the most rapid decline from 2019 to 2023, with the infection rate decreasing from 4.1% to 2.5%. Similarly, the infection rate of HPV16 exhibited a significant decrease over the same period, dropping from 4.3% in 2019 to 2.8% in 2023.

Among the LR‐HPV genotypes, HPV6 displayed the most notable downward trend, declining from 1.7% in 2019 to 0.5% in 2023. Other low‐risk types exhibited a less pronounced decline. Notably, from 2020 to 2023, HPV42, HPV43, HPV44, and HPV81 were more prevalent than the vaccine‐protected HPV6 and 11 (refer to Figure 2B).

### 3.4 Prevalence of HPV single and multiple genotype infections

**FIGURE 3:**
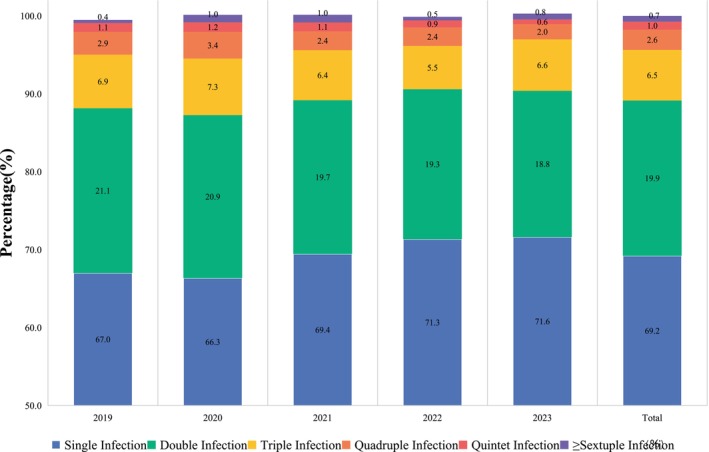
The prevalence of HPV single/multiple infection in different years. HPV, human papillomavirus.

Among the 4387 cases testing positive for HPV, 69.2% (3036/4387) exhibited single infections, while 30.8% (1351/4387) displayed multiple infections. Within the realm of multiple infections, the highest percentage, at 19.9% (875/4387), was attributed to double infections, followed by triple infections accounting for 6.5% (285/4387). The proportion of infections gradually decreased with an increase in the number of infected genotypes (refer to Figure 3). Twelve types of concurrent infections were identified in cases exhibiting multiple types of infections.

### 3.5 Prevalence of HPV according to age groups

**FIGURE 4:**
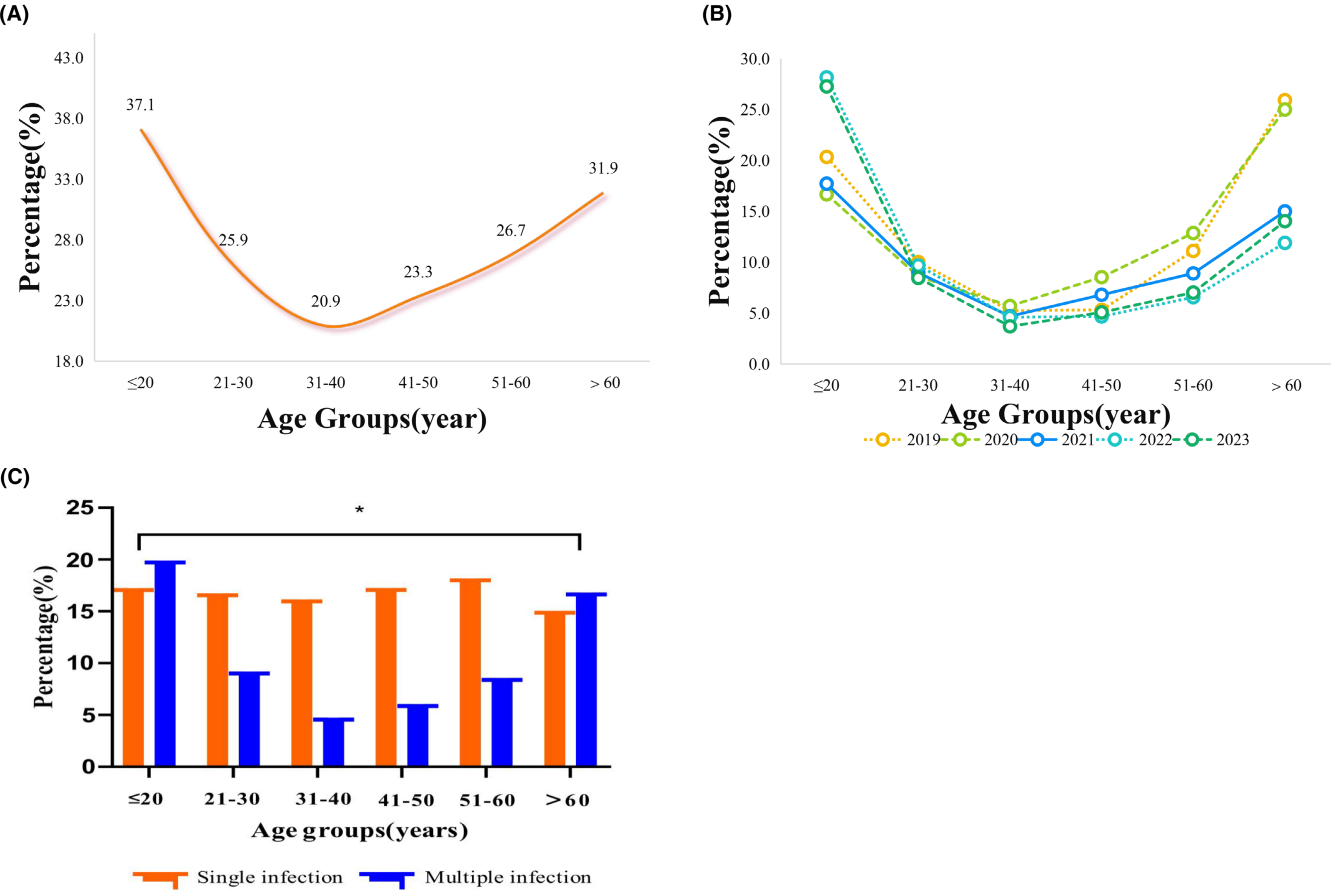
The distribution of HPV infection rate in different age groups. (A) The distribution of HPV infection rates in different age groups among 17,932 women; (B) The distribution of HPV multiple infection rates in different age groups by year; (C) The distribution of HPV single and multiple genotype infections in different age groups. HPV, human papillomavirus.

All subjects were categorized into six groups based on age (≤20, 21–30, 31–40, 41–50, 51–60, >60 years). Among the 17,923 women, the distribution of infection rates by age group displayed a “U‐shaped” pattern (see Figure 4A). The highest infection rate was observed in the age group ≤20 years at 37.1%, followed by the >60 years age group at 31.9%, and the lowest rate of 20.9% in the 31–40 years group. The distribution of multiple genotype infections across all age groups also followed a “U” shape (refer to Figure 4B), while single genotypes were not prominently evident. A statistical difference (χ^2^ = 155.0, *p* < 0.05) was observed between the rates of single and multiple genotypic infections in the six age groups (Figure 4C), with multiple infections being predominant in the ≤20 and >60 years groups.

A statistically significant difference in the positivity rate was noted between the age groups of all examined women (χ^2^ = 122.5, *p* < 0.05). From 2019 to 2023, each year exhibited statistically significant differences in positivity rates across all age groups (*p* < 0.05). Further multiple comparisons using the Bonferroni antihypertensive procedure revealed that infection rates were significantly lower in the 31–40‐year‐old group compared to the ≤20 age group (*p* < 0.05). An annual analysis of infection rates for all age groups indicated statistical differences between the 31–40 and 41–50 age groups by year (31–40, χ^2^ = 12.0, *p* < 0.05; 41–50, χ^2^ = 16.7, *p* < 0.05), with the 31–40 years group showing a decreasing trend over the years. Concurrently, the infection rate in women ≤20 years of age exhibited a tendency to increase year by year (see Table 2).

**TABLE 2:** Prevalence of human papillomavirus (HPV) infection in six age groups from 2019 to 2023.

Year	≤20	21–30	31–40^a^	41–50^#^	51–60	>60	χ^2^	*p*‐Value
	*N*, % (95% CI)	*N*, % (95% CI)	*N*, % (95% CI)	*N*, % (95% CI)	*N*, % (95% CI)	*N*, % (95% CI)		
2019	17,234.3 (27.2–41.9)	85,825.8 (22.9–28.8)	80,223.4 (20.5–26.5)^b^	80,923.6 (20.7‐26.7)	29,731.6 (26.4–37.3)	8139.5 (28.8–51.0)	23.9	<0.001
2020	12,635.7 (27.4–44.7)	67,426.6 (23.3–30.1)	80,722.9 (20.1–26.0)^c^	78,526.6 (23.6‐29.9)	30,328.4 (23.4–33.8)	6442.2 (29.9–55.2)	19.4	0.002
2021	23,735.0 (29.0–41.5)	112,726.3 (23.7–28.9)	144,921.5 (19.4–23.7)^d^	144,125.3 (23.0‐27.6)	78,627.0 (23.9–30.2)	18,030.6 (23.9–37.8)	27.0	<0.001
2022	7145.1 (33.2–57.3)	70,227.8 (24.5–31.3)	108,819.1 (16.8–21.6)^e^	115,620.3 (18.0‐22.8)	70,125.2 (22.1–28.6)	12,628.6 (20.9–37.3)	46.8	<0.001
2023	3354.5 (36.4–71.9)	54,422.4 (19.0–26.2)	94,518.1 (15.7–20.7)^f^	84,720.8 (18.1‐23.7)	59,824.9 (21.5–28.6)	11,426.3 (18.5–35.4)	33.9	<0.001
Total	63,937.1 (33.3–40.9)	390,525.9 (24.6–27.3)	509,120.9 (19.8–22.0)^g^	503,823.3 (22.2‐24.5)	268,526.7 (25.1–28.5)	56,531.9 (28.0–35.9)	122.5	<0.001

^#^ From 2019 to 2023, the HPV infection rate in the 41–50 age group was significantly different between each year (χ^2^ = 16.7, *p* = 0.002).

^a^ From 2019 to 2023, the HPV infection rate in the 31–40 age group was significantly different between each year (χ^2^ = 12.0, *p* = 0.02).

^b^ In 2019, the HPV infection rate in the 31–40 age group was significantly different from that in the ≤20 age group (*p* < 0.05) and the >60 age group (*p* < 0.05).

^c^ In 2020, the HPV infection rate in the 31–40 age group was significantly different from that in the ≤20 age group (*p* < 0.05) and the >60 age group (*p* < 0.05).

^d^ In 2021, the HPV infection rate in the 31–40 age group was significantly different from that in the ≤20 age group (*p* < 0.05).

^e^ In 2022, the HPV infection rate in the 31–40 age group was significantly different from that in the ≤20 age group (*p* < 0.05), the 21–30 age group (*p* < 0.05) and the 51–60 age group (*p* < 0.05).

^f^ In 2023, the HPV infection rate in the 31–40 age group was significantly different from that in the ≤20 age group (*p* < 0.05) and the 51–60 age group (*p* < 0.05).

^g^ From 2019 to 2023, the HPV infection rate in the 31–40 age group was significantly different from the other five age groups [≤20 age group (*p* < 0.05), 21–30 age group (*p* < 0.05), 41–50 age group (*p* < 0.05), 51–60 age group (*p* < 0.05), >60 age group (*p* < 0.05)].

**FIGURE 5:**
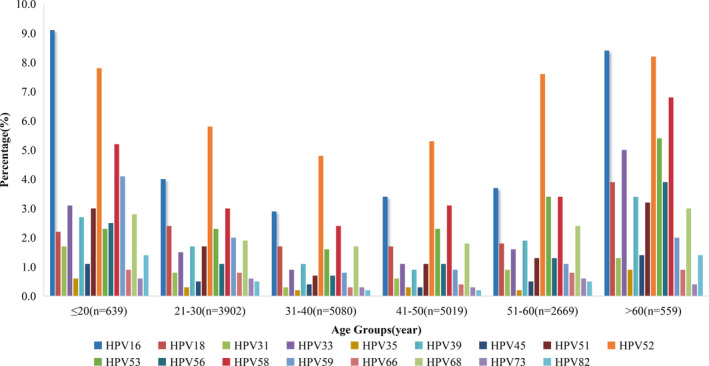
Age‐stratified distribution of 17 HR‐HPV genotypes infection. HPV, human papillomavirus.

In the analysis of the distribution of the 17 high‐risk genotypes, it was observed that the top three genotypes causing infections in each age group were HPV52, HPV16, and HPV58. HPV16 was notably prevalent in the ≤20 and >60 age groups, while HPV52 dominated in the other age groups (21–30, 31–40, 41–50, and 51–60). The 31–40 age group exhibited the lowest infection rates across most high‐risk genotypes. The ≤20 and >60 age groups displayed the highest proportion of infections among the 17 high‐risk genotypes, with the exception of HPV types 18, 53, and 73 (see Figure 5).

### 3.6 Distribution of HPV genotypes according to abnormal cytologic and histologic findings

Among the 4387 positive women in this study, 3295 underwent thin‐layer cytologic testing (TCT), leading to 265 cases diagnosed with cytologic abnormalities (8.0%). Additionally, out of 626 patients who underwent cervical biopsy, 266 were diagnosed with histologic abnormalities (42.5%). In cases, where both thin‐layer cytologic test and biopsy results were positive, single‐type infections were more prevalent than multiple types, though this difference was not statistically significant (*p* > 0.05) (refer to Table 3).

**TABLE 3:** Distribution of human papillomavirus (HPV) genotypes according to cytology/histological abnormalities.

Genotypes	ASCUS (*n* = 41) %^a^	LSIL (*n* = 134) %^a^	HSIL (*n* = 76) %^a^	AGS (*n* = 14) %^a^	Total^b^ (*n* = 265) %^a^	Genotypes	CIN1 (*n* = 103) %^a^	CIN2 (*n* = 51) %^a^	CIN3 (*n* = 73) %^a^	CA (*n* = 39) %^a^	Total^c^ (*n* = 266) %^a^
Single infection^d^	73.2	60.4	64.5	78.6	64.5	Single infection^e^	58.3	64.7	65.8	79.5	64.7
Multiple infection^f^	26.8	39.6	35.5	21.4	35.5	Multiple infection^g^	41.7	35.3	34.2	25.6	36.1
HR‐HPV											
HPV16	26.8	23.1	43.4	42.9	30.6	HPV16	26.2	37.3	63.0	64.1	44.0
HPV18	9.8	3.0	7.9	7.1	5.7	HPV18	7.8	5.9	8.2	20.5	9.4
HPV31	4.9	4.5	5.3	7.1	4.9	HPV31	1.9	2.0	0.0	5.1	1.9
HPV33	9.8	7.5	14.5	0.0	9.4	HPV33	11.7	9.8	15.1	7.7	11.7
HPV35	0.0	1.5	1.3	0.0	1.1	HPV35	1.9	3.9	1.4	0.0	1.9
HPV39	2.4	3.7	2.6	0.0	3.0	HPV39	4.9	0.0	0.0	0.0	1.9
HPV45	2.4	1.5	6.6	0.0	3.0	HPV45	1.9	3.9	1.4	2.6	2.3
HPV51	0.0	9.0	2.6	0.0	5.3	HPV51	10.7	11.8	4.1	2.6	7.5
HPV52	31.7	31.3	32.9	35.7	32.1	HPV52	40.8	37.3	21.9	10.3	30.5
HPV53	9.8	5.2	6.6	7.1	6.4	HPV53	4.9	11.8	1.4	2.6	4.9
HPV56	4.9	5.2	1.3	0.0	3.8	HPV56	2.9	7.8	1.4	2.6	3.4
HPV58	19.5	21.6	14.5	14.3	18.9	HPV58	19.4	21.6	20.5	15.4	19.5
HPV59	0.0	2.2	3.9	0.0	2.3	HPV59	6.8	3.9	4.1	2.6	4.9
HPV66	4.9	2.2	1.3	0.0	2.3	HPV66	5.8	2.0	0.0	0.0	2.6
HPV68	4.9	9.0	1.3	14.3	6.4	HPV68	6.8	3.9	1.4	0.0	3.8
HPV73	0.0	0.7	0.0	0.0	0.4	HPV73	3.9	0.0	0.0	0.0	1.5
HPV82	0.0	1.5	0.0	0.0	0.8	HPV82	0.0	0.0	2.7	0.0	0.8
LR‐HPV											
HPV6	0.0	2.2	2.6	0.0	1.9	HPV6	1.0	0.0	0.0	0.0	0.4
HPV11	2.4	5.2	1.3	0.0	3.4	HPV11	4.9	0.0	4.1	0.0	3.0
HPV42	4.9	6.0	7.9	0.0	6.0	HPV42	6.8	2.0	6.8	2.6	5.3
HPV43	4.9	4.5	1.3	0.0	3.4	HPV43	3.9	2.0	1.4	0.0	2.3
HPV44	9.8	5.2	2.6	7.1	5.3	HPV44	3.9	9.8	6.8	0.0	5.3
HPV81	4.9	7.5	1.3	0.0	4.9	HPV81	3.9	5.9	6.8	0.0	4.5

^a^ Percentage of genotypes infected in ASCUS/LSIL/HSIL/AGS/CIN1/CIN2/CIN3/CA group.

^b^ Total women with cytological abnormalities(ASCUS+LSIL+HSIL+AGS).

^c^ Total women with histological abnormalities(CIN1 + CIN2 + CIN3 + CA).

^d^ Percentage of single infection in ASCUS/LSIL/HSIL/AGS group.

^e^ Percentage of single infection in CIN1/CIN2/CIN3/CA group.

^f^ Percentage of multiple infection in ASCUS/LSIL/HSIL/AGS group.

^g^ Percentage of multiple infection in CIN1/CIN2/CIN3/CA group.

In the group with cytologic abnormalities, ASCUS accounted for 1.2% (41/3295) of the total number of tests, LSIL for 4.1% (134/3295), HSIL for 2.3% (76/3295), and AGC for 0.4% (14/3295). The three most common high‐risk genotypes in the cytologic abnormality group were HPV52 (32.1%), HPV16 (30.6%), and HPV58 (18.9%). HPV52 predominated in the ASCUS and LSIL groups, while HPV16 was predominant in the HSIL and AGC groups. HPV68 also featured among the top three high‐risk genotypes in prevalence in the AGC group (see Table 3).

In the histologic abnormality group, the three most common high‐risk genotypes were HPV16 (44.0%), HPV52 (30.5%), and HPV58 (19.5%). HPV52 predominated in CIN1, HPV52 and HPV16 were prevalent in CIN2, and HPV16 dominated in both CIN3 and CA groups (see Table 3).

The study data also revealed that the prevalence of HPV53 (6.4%), HPV68 (6.4%), and HPV51 (5.3%) in cytologic abnormalities results exceeded that of the vaccine‐protected types HPV31 (4.9%) and HPV45 (3.0%). Similarly, in histologic abnormalities results, the rates of HPV51 (7.5%), HPV53 (4.9%)/HPV59 (4.9%), and HPV68 (3.8%) were higher than HPV31 (1.9%) and HPV45 (2.3%) (see Table 3).

In the group of CIN 2–3 and CA, there were 51.6% and 74.4% of high‐risk HPV single type, also 38.7% and 30.8% of multiple infections, respectively. The single infection of HR‐HPV subtype covered by the nine‐valent vaccine was detected in 45.2% and 69.2% of the 2 groups, respectively, with the multiple infections were 33.1% and 28.2%, respectively. HPV16 was the most prevalent in both groups, at 40.3% and 64.1%, respectively, and was more prevalent in single infection than in multiple infections (24.2% vs. 16.1% for CIN 2–3, 41.0% vs. 23.1% for CA). Mono‐infection with HPV18 was predominant in cervical cancer patients (17.9%), while it was uncommon in CIN2‐3 (0.8%). No infected with HPV45 and HPV52 was found in cervical cancer group in this study. Meanwhile, the rate of infection of HN9‐HPV(high‐risk non‐nine‐valent vaccine covering HPV subtypes) was 12.1% (15/124) and 2.6% (1/39), of which the single infection caused 6.5% of CIN2‐3 and 2.6% of CA.HPV51 caused 4.8% of CIN2‐3, of which 2.4% were isolated infections.HPV35 caused 3.2% of CIN2‐3 and 2.4% of infections alone.HPV59 accounted for 1.6% of CIN2‐3 and 2.6% of CA alone. And there was also a 1.6% share of CIN2‐3 due to HPV53.Among 16 patients infected with LN9‐HPV (low‐risk non‐nine‐valent vaccine covering HPV subtypes), the infection rates were 12.9% for CIN2‐3.And the number of patients experiencing CIN2‐3 solely due to HPV42 infection exceeded that of other low‐risk HPV types (Table 4).

**TABLE 4:** Infection of different types of human papillomavirus (HPV) in patients with CIN 2–3 and cervical cancer.

Genotypes	CIN2‐3 (*n* = 124)		CA (*n* = 39)	
	*n*	%	*n*	%
HR‐HPV				
Single type	64	51.6	29	74.4
Multiple infections	48	38.7	12	30.8
9‐HRHPV				
HPV16				
Single type	30	24.2	16	41.0
Multiple infections	20	16.1	9	23.1
HPV18				
Single type	1	0.8	7	17.9
Multiple infections	7	5.6	1	2.6
HPV31				
Single type	0	0.0	1	2.6
Multiple infections^a^	0	0.0	0	0.0
HPV33				
Single type	4	3.2	1	2.6
Multiple infections^a^	3	2.4	1	2.6
HPV45				
Single type	0	0.0	0	0.0
Multiple infections^a^	1	0.8	0	0.0
HPV52				
Single type	10	8.1	0	0.0
Multiple infections^a^	7	5.6	0	0.0
HPV58				
Single type	11	8.9	2	5.1
Multiple infections^a^	3	2.4	1	2.6
Total				
Single type	56	45.2	27	69.2
Multiple infections	41	33.1	11	28.2
HN9‐HPV				
HPV35				
Single type	3	2.4	0	0.0
Multiple infections^a^	1	0.8	0	0.0
HPV39				
Single type	0	0.0	0	0.0
Multiple infections^a^	0	0.0	0	0.0
HPV51				
Single type	3	2.4	0	0.0
Multiple infections^a^	3	2.4	0	0.0
HPV53				
Single type	1	0.8	0	0.0
Multiple infections^a^	1	0.8	0	0.0
HPV56				
Single type	0	0.0	0	0.0
Multiple infections^a^	0	0.0	0	0.0
HPV59				
Single type	0	0.0	1	2.6
Multiple infections^a^	2	1.6	0	0.0
HPV66				
Single type	0	0.0	0	0.0
Multiple infections^a^	0	0.0	0	0.0
HPV68				
Single type	0	0.0	0	0.0
Multiple infections^a^	0	0.0	0	0.0
HPV73				
Single type	0	0.0	0	0.0
Multiple infections^a^	0	0.0	0	0.0
HPV82				
Single type	1	0.8	0	0.0
Multiple infections^a^	0	0.0	0	0.0
Total				
Single type	8	6.5	1	2.6
Multiple infections	7	5.6	0	0.0
LR‐HPV				
Single type	16	12.9	0	0.0
9‐LRHPV				
HPV6				
Single type	0	0.0	0	0.0
HPV11				
Single type	2	1.6	0	0.0
LN9‐HPV				
HPV42				
Single type	8	6.5	0	0.0
HPV43				
Single type	1	0.8	0	0.0
HPV44				
Single type	3	2.4	0	0.0
HPV81				
Single type	2	1.6	0	0.0

*Note*: 9‐HRHPV, high‐risk human papillomavirus covered by the nine‐valent vaccine (including 16, 18, 31, 33, 45, 52, and 58 type); HN9‐HPV, high‐risk human papillomavirus covered by the non‐nine‐valent vaccine (including 35, 39, 51, 53, 56,59, 66,68,73, and 82 type); Multiple infections^a^, 2 or more HPV subtypes, excluding HPV16 and/or HPV18; 9‐LRHPV, low‐risk human papillomavirus covered by the nine‐valent vaccine (including 6 and 11 type); LN9‐HPV, low‐risk human papillomavirus covered by the non‐nine‐valent vaccine (including 42, 43, 44, and 81 type).

Notably, HPV‐positive patients aged <60 years predominantly exhibited LSIL in the cytologic abnormality group, while those aged >60 years predominantly displayed HSIL (Table 5). CIN1 was predominant in the histologic abnormality group among those <60 years of age, whereas CA was prevalent among those >60 years of age. Interestingly, we found that HPV‐positive patients aged 31–40 years reached the highest proportion of 29.0% in the prevalence of CIN2‐3, while the overall prevalence of HPV infection was lowest in this age group, compared with other age groups. (Table 6).

**TABLE 5:** Age distribution of prevalence of human papillomavirus‐positive patients in ASCUS, LSIL, HSIL, and AGS.

Cytological abnormalities	≤20 (*n* = 133) %	21–30 (*n* = 775) %	31–40 (*n* = 860) %	41–50 (*n* = 841) %	51–60 (*n* = 545) %	>60 (*n* = 141) %	Total (*n* = 3295) %
ASCUS	0.0	0.8	1.6	1.2	1.3	2.8	1.2
LSIL	4.5	3.9	4.0	3.9	5.1	2.1	4.1
HSIL	0.0	1.2	2.9	1.9	2.8	7.8	2.3
AGS	0.0	0.3	0.5	0.6	0.6	0.0	0.4
Total	4.5	6.1	9.0	7.6	9.7	12.8	8.0

**TABLE 6:** Age distribution of prevalence of human papillomavirus‐positive patients in CIN1, CIN2, CIN3, and CA.

Histological abnormalities	≤20 (*n* = 11) %	21–30 (*n* = 84) %	31–40 (*n* = 138) %	41–50 (*n* = 214) %	51–60 (*n* = 141) %	>60 (*n* = 38) %	Total (*n* = 626) %
CIN1	18.2	19.0	15.9	17.3	17.7	2.6	16.5
CIN2	0.0	7.1	13.8	6.5	7.1	5.3	8.1
CIN3	0.0	16.7	15.2	7.5	10.6	18.4	11.7
CIN2‐3^a^	0.0	23.8	29.0	14.0	17.7	23.7	19.8
CA	0.0	0.0	2.2	5.1	10.6	26.3	6.2
Total	18.2	45.2	45.7	36.4	46.1	52.6	42.5

^a^ CIN2‐3: CIN2 and CIN3.

## 4 DISCUSSION

This is a 5‐year cross‐sectional pooled epidemiologic study of HPV infection in women in Sichuan, China. Our analysis revealed an overall HPV infection rate of 24.5%, significantly higher in the outpatient group than in the physical examination group. The most common types of infections were HPV52, 16, 58, 53, 68, and 18. The age distribution of HPV exhibited a U‐shaped pattern, with the 31–40 age group having the lowest prevalence of infection but the highest prevalence of the precancerous lesion CIN2‐3. Notably, from 2019 to 2023, the HPV infection rate exhibited a decreasing trend, and certain high‐risk types, particularly HPV16 and HPV58, experienced significant declines. We discovered that HPV51, HPV35, HPV59, and HPV53 emerged as the predominant non‐vaccine high‐risk genotypes that primarily contribute to the development of CIN2‐3 and CA.

The prevalence of HPV infection and the distribution of HPV genotypes varied significantly among different regions and populations. The overall infection rate (24.5%) was considerably lower than previously reported in China (50.64%)^29^ but aligned with studies by Luo^18^ and Li^30^ in Sichuan. Additionally, the HPV infection rate in the outpatient group (26.5%) was significantly higher than that in the physical examination group (17.5%), consistent with the findings of Zhang.^31^ The reason for the substantial difference is mainly due to the outpatients usually come to a hospital to address medical issues, including cervical intraepithelial neoplasia, genital warts, and other diseases, their HPV prevalence is higher than the women who participate in routine physical examinations.^32^ Compared to other regions of China, our physical examination infection rates were generally in line with previous results from Sichuan (15.29%), Guangdong (17.25%), Fujian (16.2%), and Guangxi (19.5%) in the southern region.^18^, ^33^, ^34^, ^35^ However, they were higher than those in Beijing (11.9%), Zhejiang (13.6%), and Nanjing (10.6%).^31^, ^32^, ^36^ These disparities may be attributed to differences in study populations, socioeconomics, literacy, and health awareness between the northern and southern regions. Therefore, continuous attention to HPV infection population and in access to adequate screening is imperative in Sichuan to enhance social mobilization for cervical cancer prevention and treatment.

Significant disparities were also observed in the distribution of targeted HPV genotypes across different regions. For instance, HR‐HPV was widespread globally, with prevalent HPV genotypes in Italy being HPV16, HPV31, HPV66, HPV59, and HPV51,^37^ and in the western part of Brazil being HPV18, HPV16, HPV31, HPV58, HPV33, and HPV45.^38^ Our study identified the most common genotypes as HPV52, 16, 58, 53, 68, and 18, with HPV52 exhibiting the highest prevalence of infection. This aligns with recent findings on HPV genotype data from 14 provinces in China, indicating that the top three most prevalent genotypes in China are HPV52, HPV16, and HPV58.^31^ Further analysis of genotype distribution in the cytology/histology abnormal group revealed that the most common HR‐HPV types were dominated by HPV52, HPV16, and HPV58. However, HPV16 predominantly appeared in high‐grade intraepithelial neoplasia and carcinoma in situ, constituting over 60% of cases. Notably, no cases of cervical cancer were solely attributed to HPV52 infection, and HPV52 infection was absent in the cellular/tissue abnormalities population over 60 years old. This consistency with existing studies suggesting the rarity of HPV52 in cervical cancer^39^ raises the possibility that the prevalence of HPV52 could be mitigated through increased vaccination with the 9v‐HPV vaccine or by expanding the age range for 9v‐HPV vaccine (A measure approved in China on August 30, 2022, extending the age for 9v‐HPV vaccine from 16 to 26 years to 9 to 45 years.).

Our data also revealed that HPV53 and HPV68 stand out as the two high‐risk types exhibiting the highest infection rates among non‐vaccine‐protected genotypes. Notably, HPV68 has been underreported previously. Yet, a comprehensive study on HPV prevalence in Sichuan province^30^ and a recent large‐scale cross‐sectional survey involving 38,056 samples^40^ demonstrated a notable prevalence of HPV68. One study manifested women infected with HPV68 had a 5.78 times higher risk of CA than HPV‐negative women.^41^ HPV68 (3.8%) accounted for the top four non‐vaccine type infections in this histologic abnormality group, as did HPV51 (7.5), HPV53 (4.9), and HPV59 (4.9), all of which were higher than the vaccine‐type HPV31 (1.9%) and HPV45 (2.2%). We further analyzed the impact of non‐vaccine high‐risk types in histopathology. It was found that only infection with non‐nine‐valent vaccine types (HPV35, HPV39, HPV51, HPV53, HPV56, HPV59, HPV66, HPV68, HPV73, and HPV82) caused 9.1% of CIN2+ (including CIN2, CIN3 and CA). Among them, the HN9‐HPV infection rates in patients with cervical CIN 2–3 and cervical cancer were 6.5% and 2.6%, respectively, which was significantly higher than the study of Ma MJ et al (4.3% and 1.5%, respectively).^22^ Meanwhile, our findings revealed that HPV51, HPV35, HPV59, and HPV53 constituted the predominant HN9‐HPV genotypes that were primarily associated with the development of CIN2‐3 and CA. This is consistent with the increased data on cervical lesions and cervical cancer caused by non‐vaccine types in some studies. For example, Gargano et al. found a significant increase in CIN2+ attributable to non‐vaccine types (including 39, 45, 51, 52, 58, and 59) among 25–29 years old post quadrivalent HPV vaccination,^42^ and Ruan et al. found that HPV types 39, 53, 59, 66, and 68, which are not covered by the nine‐valent HPV vaccine, accounted for 8.25% of the cervical cancer.^41^ And these genotypes have the same cellular pathway as HR‐HPV such as HPV16, 18, promoting the development of cervical cancer.^8^, ^9^, ^43^ These data suggest that the nine‐valent vaccine might not offer protection against all HPV‐related infections and diseases beyond its specific vaccine types.^44^, ^45^ Therefore, it is imperative to remain vigilant regarding cervical lesions and cervical cancer resulting from non‐vaccine HPV types in the post‐vaccination era. This underscores the need for the development of HPV vaccines that encompass a broader range of carcinogenic genotypes, thereby enhancing their protective efficacy.^22^

The distribution of age plays a pivotal role in cervical cancer screening and vaccine effectiveness. Numerous studies, particularly focusing on young and elderly women, have identified a “U”‐shaped pattern in the distribution of HPV infection within these age groups.^12^, ^18^, ^46^ Our study aligns with this pattern, noting the highest infection prevalence at ≤20 years of age, followed by >60 years of age. This overall “U”‐shaped prevalence and the percentage of multiple infections among age groups suggest various factors. First, weakened immunity, attributed to cervical injuries during early sexual activity and riskier deliveries in younger women, potentially exposes them to prolonged HPV infections, influencing their immune systems.^31^, ^47^ Similarly, older women may experience compromised immune competence due to hormonal changes during menopause, leading to increased HPV infections through viral persistence or reactivation of latent infections.^48^ Second, the relatively low number of tests in both age groups, particularly in the elderly population, which has a higher susceptibility to cervical cancer, could be due to inadequate health knowledge and hygiene awareness.^49^ Recognizing this, China has extended the age of health screening for this population from 65 to 69 years.^50^ Third, the low vaccination rate in China, merely 2.6%–11.0%^31^ compared to 54.5%^51^ in the United States, may contribute to the predominant HPV16 infections in both ≤20 and >60 age groups, despite the introduction of the vaccine in Sichuan since 2018. Reasons for the low vaccination rate include vaccine cost, concerns about adverse events,^52^ and its exclusion from the national immunization program. Therefore, we advocate for increased attention to both younger and older age groups to optimize age‐based screening and vaccination strategies, potentially mitigating the risk of cervical cancer development.

We observed that, although the 31–40‐year‐old group exhibited the lowest prevalence of HPV infection, it recorded the highest percentage in the prevalence of CIN1‐3 compared to other age groups. Notably, we identified two cases of cervical cancer in 31‐year‐olds and one in 37‐year‐olds. This underscores the importance of prioritizing HPV screening in this age group within Sichuan. Once HPV infection is detected in women of this age group, incorporating various tests to rule out or confirm diseases may be considered to enhance prevention and mitigate the progression of cervical diseases.

In this study, the infection rate exhibited a consistent yearly decrease from 2019 to 2023, aligning with findings from a study in Suzhou.^53^ Among different populations, the physical examination group displayed a declining trend over the years. The infection rates for women aged 31–40 and 51–60 also decreased, while those for women ≤20 years old increased. Notably, there was a decreasing trend in HR‐HPV genotypes 16, 31, 35, 39, 56, 58, and 73, with HPV16 and HPV58 experiencing the most substantial decrease. The decline in HPV6 was most pronounced in LR‐HPV genotypes, and even isolated infections were not found in our CIN2‐3 and CA groups. This decline could be attributed to the protective effect of prior vaccination coverage and the reduction in unnecessary sexual activity due to quarantine measures amid the ongoing COVID‐19 outbreak.^12^ A meta‐analysis reported significant changes in the prevalence of HPV genotypes within 4 years of vaccine introduction and 5–8 years after vaccination.^54^ A Japanese study revealed that among women aged 18–24 years with high vaccine coverage (68.2%), the prevalence of HPV16, 18 and high‐risk HPV types decreased from 36.7% and 69.4% before vaccination to 5.8% and 50.0% after vaccination, respectively.^55^ And a Swedish study also showed that implementing both HPV vaccination and HPV screening would reduce the incidence of HPV 16 from 9.1 (per 100 woman‐years) in 2020 to 3.6 (born 1994–1998) in 2024 if the trial had a 30% participation rate.^56^ Concurrently, certain non‐vaccine‐covered HPV types, namely 51, 56, 68, and 44, exhibited an increasing trend, consistent with a recent meta‐analysis^21^ indicating rising infection rates of non‐vaccine‐covered types in the post‐vaccine era. Ongoing data collection will enable us to monitor the trend of HPV genotype distribution in future studies.

However, several limitations should be acknowledged. First, we were unable to confirm whether these patients had received the HPV vaccine, as vaccination can influence the outcomes of the targeted genotypes. Second, the lack of data from other areas of Sichuan province may limit the generalizability of our findings to all women in the province. Third, the absence of demographic and habitual characteristics, such as smoking history and number of sexual partners, prevented an assessment of their impact on HPV prevalence. Nonetheless, our data offer valuable insights for unvaccinated and unscreened women in Sichuan.

## 5 CONCLUSIONS

In summary, our study is predicated on the most extensive study of HPV infection prevalence based on 5 years in the post‐vaccination era in Sichuan. The study revealed an overall HPV infection prevalence of 24.5%, primarily associated with high prevalence of HPV genotypes 52, 16, 58, 53, 68, and 18. The age distribution of HPV infection exhibited a “U” shape, with the lowest prevalence in women aged 31–40; however, this group displayed the highest prevalence of precancerous lesions CIN2‐3. HPV16 predominated in high‐grade intraepithelial neoplasia and carcinoma in situ, while HPV type 52 was not individually detected in cervical cancer cases. Several non‐vaccine‐covered HPV subtypes, including HPV35, 51, 53, 59, and 68, also exhibited high prevalence. Infection rates demonstrated a declining trend from 2019 to 2023, with HPV16 and HPV58 displaying the most rapid rates of decline. These findings offer valuable insights for assessing the optimization of cervical cancer screening and vaccination programs in Sichuan, China.

## AUTHOR CONTRIBUTIONS

**Bangzhu Mo:** Data curation (lead); writing – original draft (lead). **Yuanxin Ye:** Writing – original draft (equal); writing – review and editing (equal). **Maowen Yu:** Supervision (equal). **Xianli Tong:** Formal analysis (equal). **Hongmei Cao:** Data curation (equal). **Chunmei Du:** Formal analysis (equal). **Jiangrong Luo:** Conceptualization (equal); supervision (equal). **Chunbao Xie:** Conceptualization (lead); writing – review and editing (lead).

## FUNDING INFORMATION

This study was supported by Sichuan Science and Technology Program (2023YFS0086) and Jintang County Medical Research Program (2023011).

## CONFLICT OF INTEREST STATEMENT

Authors state no conflict of interest.

## ETHICS STATEMENT

Ethical approval for the study was obtained from the Ethics Committee of the Jintang First People's Hospital (approval number: 20231017036).

## Supporting information


Table S1.

## Data Availability

The data that support the findings of this study are available from the corresponding author on a reasonable request.
