# Supplementary material for: Prevalence and genotype distribution of HPV combined with cervical pathological results in women from Sichuan, China: A cross‐sectional study based on post‐vaccination period 2019 to 2023
Source: Cancer Med. 2024 Aug 27;13(16):e70148. doi: 10.1002/cam4.70148 (PMC11348228; doi:10.1002/cam4.70148)
Supplement: Supplementary file 1 — Table S1. [file CAM4-13-e70148-s001.docx]

| HPV type | 2019(n=3019),% | 2020(n=2759),% | 2021(n=5220),% | 2022(n=3844),% | 2023(n=3081),% | P_trend_ |
| --- | --- | --- | --- | --- | --- | --- |
| HR-HPV |  |  |  |  |  |  |
| HPV16 | 4.3 | 4.3 | 4.1 | 3.4 | 2.8 | <0.001 |
| HPV18 | 1.9 | 1.7 | 2.6 | 1.5 | 1.6 | 0.186 |
| HPV31 | 1.0 | 1.1 | 0.5 | 0.4 | 0.5 | <0.001 |
| HPV33 | 1.2 | 1.6 | 1.6 | 1.4 | 1.1 | 0.565 |
| HPV35 | 0.2 | 0.5 | 0.4 | 0.2 | 0.1 | 0.048 |
| HPV39 | 1.6 | 1.7 | 1.6 | 1.2 | 1.0 | 0.011 |
| HPV45 | 0.5 | 0.5 | 0.5 | 0.3 | 0.4 | 0.423 |
| HPV51 | 1.5 | 1.3 | 1.4 | 1.0 | 1.3 | 0.276 |
| HPV52 | 5.4 | 6.6 | 5.6 | 6.1 | 5.5 | 0.918 |
| HPV53 | 2.6 | 2.6 | 2.4 | 2.4 | 2.0 | 0.111 |
| HPV56 | 1.4 | 1.6 | 1.0 | 0.8 | 1.0 | 0.008 |
| HPV58 | 4.1 | 4.0 | 3.0 | 2.5 | 2.5 | <0.001 |
| HPV59 | 1.4 | 1.6 | 1.1 | 1.2 | 1.2 | 0.257 |
| HPV66 | 0.6 | 0.6 | 0.6 | 0.5 | 0.6 | 0.989 |
| HPV68 | 2.0 | 2.0 | 2.1 | 1.6 | 2.1 | 0.691 |
| HPV73 | 0.6 | 0.3 | 0.6 | 0.4 | 0.1 | 0.013 |
| HPV82 | 0.6 | 0.4 | 0.4 | 0.4 | 0.3 | 0.075 |
| LR-HPV |  |  |  |  |  |  |
| HPV6 | 1.7 | 1.5 | 0.8 | 0.6 | 0.5 | 0.001 |
| HPV11 | 0.9 | 0.7 | 0.7 | 0.6 | 0.6 | 0.163 |
| HPV42 | 1.7 | 2.0 | 2.1 | 2.1 | 1.5 | 0.763 |
| HPV43 | 1.4 | 1.4 | 1.3 | 1.2 | 1.0 | 0.123 |
| HPV44 | 2.1 | 2.1 | 1.4 | 1.4 | 1.7 | 0.036 |
| HPV81 | 1.5 | 1.8 | 2.4 | 1.8 | 1.9 | 0.295 |

Tables S1 Trends of prevalence of different HPV genotypes from 2019 to 2023(detail data).
